# Molecular Mechanisms of Drug Resistance in *Leishmania* spp.

**DOI:** 10.3390/pathogens13100835

**Published:** 2024-09-27

**Authors:** Maria Juliana Moncada-Diaz, Cristian Camilo Rodríguez-Almonacid, Eyson Quiceno-Giraldo, Francis T. H. Khuong, Carlos Muskus, Zemfira N. Karamysheva

**Affiliations:** 1 Department of Cell Biology and Biochemistry, Texas Tech University Health Science Center, Lubbock, TX 79430, USA; juliana.moncada@ttuhsc.edu (M.J.M.-D.); rod65531@ttuhsc.edu (C.C.R.-A.); eyson.quiceno@ttuhsc.edu (E.Q.-G.); francis.khuong@ttuhsc.edu (F.T.H.K.); 2 Department of Biological Sciences, Texas Tech University, Lubbock, TX 79409, USA; 3 Programa de Estudio y Control de Enfermedades Tropicales-PECET, Facultad de Medicina, Universidad de Antioquia, Medellín 050010, Colombia; carlos.muskus@udea.edu.co

**Keywords:** drug resistance, genomic changes, transcriptional control, translational reprograming, *Leishmania* parasites

## Abstract

The protozoan parasite *Leishmania* causes leishmaniasis, a neglected tropical disease, that disproportionately affects underdeveloped countries. This disease has major health, economic, and social implications, particularly because of the limited treatment options, high cost, the severe side effects associated with available therapeutics, and the high rate of treatment failure caused by the parasites’ growing resistance to current medications. In this review, we describe first the common strategies used by pathogens to develop drug resistance and then focus on the arsenal of available drugs to treat leishmaniasis, their modes of action, and the molecular mechanisms contributing to drug resistance in *Leishmania* spp., including the role of genomic, transcriptional, and translational control. We focus more specifically on our recent discovery of translational reprogramming as a major driver of drug resistance leading to coordinated changes in the translation of transcripts and orchestrating changes in metabolome and lipidome to support drug resistance. A thorough understanding of these mechanisms is essential to identify the key elements needed to combat resistance and improve leishmaniasis treatment methods.

## 1. Introduction

Drug resistance is a major health problem of modern times, causing serious economic losses associated with high treatment costs and a reduction in drug effectiveness, treatment failures and relapses, a higher risk of disease spread, and longer hospitalizations. This issue also poses a significant social burden associated with difficult access to treatment options for people with limited resources in underdeveloped countries, increasing morbidity and mortality as well as impacting the quality of life of patients and their families [1]. Diseases caused by drug-resistant microorganisms are a proven global challenge with decreasing rates of successful treatments, limiting available options and potentially leading to untreatable disease [2]. These resistant infections can be caused by several factors related to the microorganism itself, such as its genetic diversity, horizontal gene transfer, and genetic mutations induced by the environment that cause aneuploidies and polygenic resistance phenotypes [3]. Likewise, other factors related to the human host such as immune system disorders, as well as the misuse of antimicrobial drugs during medical procedures and their dissemination in the environment by the agricultural and veterinary industry, contribute significantly to drug resistance [3,4].

Another mechanism allowing pathogen survival despite drug treatment is through persistence. It is important to emphasize that the mechanisms of drug resistance and persistence are different. Persisters survive therapeutic intervention by increasing their tolerance via entering a dormant state and evading host cell defenses. There are typically no genetic changes in persisters and when drug pressure is gone, they may return to the proliferative state, sensitive to the drug [5]. Both resistance and persistence are mechanisms developed by microorganisms to ensure survival in the host environment, and although they are different, it has been suggested that persistent cells may be precursors to resistance mechanisms [6].

Several common strategies have been reported in the development of drug resistance in bacteria, fungi, protozoan parasites, and even in human cancer cells [7,8]. These strategies fall into four main categories: (i) limiting drug uptake; (ii) increasing drug efflux; (iii) drug inactivation; and (iv) drug target modification. Decreased uptake and an increased efflux of antibiotic drugs through membrane pumps are the most well-known mechanisms that lead to multidrug resistance in different microorganisms, especially in bacteria [9,10]. Efflux pumps are transmembrane proteins that can recognize and expel a variety of toxic chemicals and antimicrobial compounds from the cell, and are used to reduce the level of cellular concentrations of antibiotics to a minimum level that would not affect their survival and multiplication [10]. Other common mechanisms in bacteria are directly related to the target sites: the modification of the target, its substitution with new targets with similar functions, the protection of target sites such as ribosomal protection proteins (RPPs), and the massive production of the target, exceeding the antibiotic’s capacity [11]. Similar to bacteria, the general mechanisms of antifungal resistance include modifications in the drug binding site, efflux pumps, and drug inactivation [8]. Likewise, mutations in genes involved in ergosterol synthesis and glucan synthases, as well as the positive regulation during the translation of genes encoding efflux pumps proteins, confer resistance to antifungal drugs. For instance, mutations in single genes such as ERG11 and pectin degradation regulator-1 (PDR-1), along with the positive regulation of genes (ERG5, ERG6 and ERG25), associated with ergosterol biosynthesis, are associated with amphotericin B (AmB) resistance [3,4,12]. Cancer cells have also developed various drug resistance mechanisms similar to those used by bacteria and other eukaryotes; these mechanisms involve alterations at genomic, transcriptional, translational, and metabolomic levels [13]. Genetic alterations conferring drug resistance include mutations, deletions, rearrangements, and translocations [13,14,15]. At the translational level, the overexpression of efflux pumps and alterations in signaling pathways are significant contributors to resistance [16,17]; moreover, translational reprograming allows cancer cells to modify their protein synthesis machinery to evade the effects of therapeutic agents, influencing metabolic processes that support cellular proliferation, survival, and resistance to death [18,19].

Resistance mechanisms have been substantially less studied in pathogenic protozoa parasites of the Trypanosomatidae family. In general, protozoa parasites also have the machinery necessary to limit uptake, inactivate, or increase the active efflux of a drug, as well as the tools to modify a drug’s target. The principles are similar to other pathogens such as bacteria, fungi, and other protozoa as well as cancer cells. It has been shown that the loss of membrane transporters could generate resistance to treatment due to the reduction in drug accumulation in parasitic cells [20]. For instance, in *Trypanosoma brucei*, the functional loss of membrane transporters such as aminopurine transporter P2 encoded by the gene TbAT1 and high-affinity pentamidine transporter (HAPT1), lead to cross-resistance to melarsoprol and pentamidine (PTM) drugs, used to treat human African trypanosomiasis [20].

**Figure 1:**
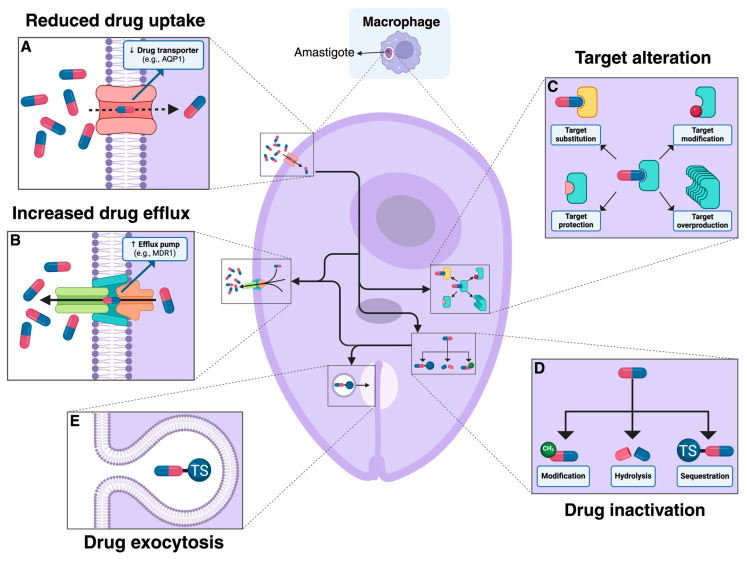
Drug-resistance mechanisms employed by amastigotes of *Leishmania* spp. (**A**) Deletion or reduced expression of drug transporters such as aquaglyceroporin 1 (AQP1) can diminish cellular drug uptake. (**B**) The overexpression of ABC transporters like MDR1, ABCI4, ABCG4 or ABCG6 helps the parasite efflux the drug and diminish its effect [25]. (**C**) Target alteration involves (i) the modification of the target to reduce drug binding, (ii) the substitution of the target by a new protein with a similar function that is not inhibited by the drug, (iii) the association of a target protection protein with the target, and iv) target overproduction to compensate for the drug’s inhibitory effect [26,27]. (**D**) Drug inactivation can occur through modification, hydrolysis, or the sequestration of the drug, rendering it ineffective [25,26]. (**E**) Drug exocytosis involves the encapsulation and expulsion of the drug or its conjugates from the parasite cell, usually thorough the flagellar pocket [25]. These mechanisms collectively enable the parasite to evade the therapeutic effects of drugs and persist in the host.

Similar to previously described models, the mechanisms of acquired resistance in *Leishmania* species are associated with the up-regulation of proteins that degrade or reduce the toxic effects of these drugs, decrease drug entry, and increase export via transporters [21] (Figure 1). For instance, the deletion or reduced expression of aquaglyceroporin 1 (AQP1) renders parasites resistant to trivalent antimony (Sb^III^) [22,23]. The overexpression of ATP binding cassette (ABC) transporters involved in transmembrane ATP-dependent transport confers resistance associated with vesicle sequestration; while another type of ABC transporters like the pentamidine resistance protein (PRP1) has been related to the resistance to pentamidine (PTM). The molecular mechanisms of resistance in *Leishmania* have been primarily described for antimonials, but it is also important to understand the mechanisms that confer resistance to other drugs used in the treatment of leishmaniasis, especially considering combination therapies to which parasites can also develop resistance [24].

The mechanisms of resistance in *Leishmania* have not been explored in depth, especially during translation. There is little information available about translation profiles during infection and the transition to the host environment; however, there is evidence for the important role of translational reprogramming as a major driver of drug resistance mechanisms [28]. Recurrent treatment failures, long-term infections, limited treatment alternatives, reports of persistence, and drug resistance mechanisms that could rapidly evolve during the treatment of leishmaniasis have motivated us to further explore what is known about the molecular mechanisms of drug resistance. Understanding the mechanisms associated with molecular changes during resistance could enhance the approach to treatments, predict the susceptibility of strains from clinical specimens to a drug, thereby extending the half-life of the drug, improve the development and efficacy of new drugs, and ultimately reduce the spread of resistant strains leading to the adequate control of leishmaniasis disease [29].

## 2. Available Treatments against Leishmaniasis

**Figure 2:**
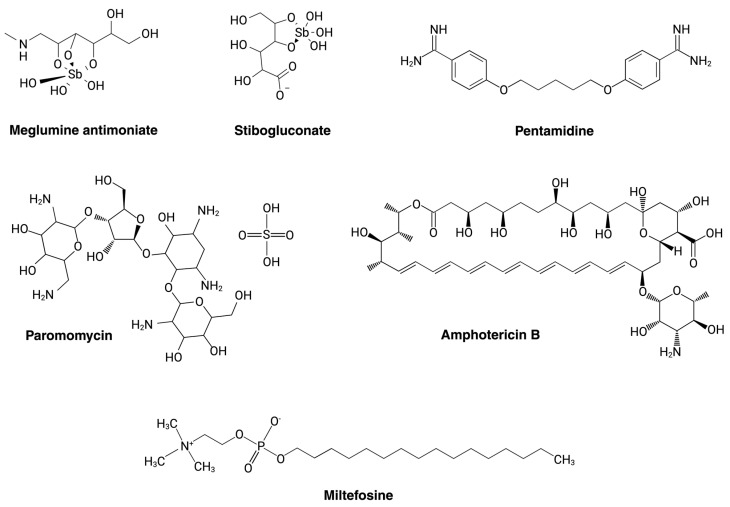
Commonly used drugs for leishmaniasis. Meglumine antimoniate and stibogluconate are pentavalent antimonials used to treat cutaneous (CL) [36,37], mucocutaneous (ML) [38], and visceral leishmaniasis (VL) [38]. Pentamidine is also employed for the treatment of all three forms of this disease [39,40,41]. Paromomycin and miltefosine have shown effectiveness in treating both VL [42] and CL [43,44,45]. Amphotericin B is also an effective treatment option for VL [46] and CL [47].

Leishmaniasis is a neglected tropical disease (NTD) caused by several species of the protozoan parasite *Leishmania*. These parasites are digenetic, and acquire their infectivity inside a phlebotomine vector, where the procyclic promastigote passes through several stages before developing into its infectious form as a metacyclic promastigote. Subsequent introduction into the bloodstream of the human host through the sandfly’s bite then allows the parasite to establish itself inside host macrophages and differentiate into intracellular amastigotes [30]. The disease represents a significant public health issue and can occur in three different forms: cutaneous leishmaniasis (CL), mucocutaneous leishmaniasis (MCL), and visceral leishmaniasis (VL), with the last being the most lethal [31]. Various drugs have been used over the years to treat the different manifestations of this disease. Current treatment regimens are individualized, and their success is related to the clinical manifestations and other patient-associated factors [32]. Some of the most common treatments approved by the Food and Drug Administration (FDA) include pentavalent antimony (Sb^V^), miltefosine (MLT), amphotericin B (AmB), paromomycin (PMM), pentamidine (PTM), and others [33] (Figure 2). Although the mechanisms of action of these commercially available treatments have not been fully described for *Leishmania* spp., it is known that PMM may interfere with the cellular energy metabolism and can induce respiratory dysfunction in *Leishmania* parasites [34]. MLT and Sb^V^ could affect the mitochondria, AmB could alter the permeability of the cell membrane, and PTM and PMM are associated with DNA damage and the inhibition of RNA and protein synthesis, all culminating in the death of the parasitic cells [35]. The modes of action of the most-used drugs to treat leishmaniasis are described in Table 1.

First-line prescription drugs including Sb^V^ have remained the mainstay of treatment for visceral leishmaniasis in recent years; however, they have limitations related to significant adverse effects such as liver, renal, and cardiac toxicity as well as other gastrointestinal symptoms associated with systemic administration [59]. To reduce some of these side effects, the Pan American Health Organization has approved the intralesional administration as an acceptable alternative [60]. The ability to reduce Sb^V^ to Sb^III^ has been reported as a mechanism of resistance, especially in the intracellular amastigotes of *Leishmania* [61]; other mechanisms have also been described, such as drug sequestration, efflux, and the increase in intracellular thiol levels as a defense mechanism to combat the oxidative stress generated by antimonials [21,62,63]. MLT is a chemotherapeutic agent included in the World Health Organization (WHO)’s list of essential medicines as a first-line treatment option for cutaneous and mucocutaneous leishmaniasis caused by New World species of *Leishmania (Viannia) braziliensis, L. (V.) panamensis*, and *L. (V.) guyanensis* in Latin American countries, such as Brazil, Colombia, Guatemala, and Peru [64]. This pharmaceutical agent induces an alteration in Ca^2+^ homeostasis [50], resulting in increased an intracellular Ca^2+^ concentration and contributing to the death of the parasite. Clinical studies have documented renal and hepatic toxicity [65], as well as other drug-related drawbacks such as effects on the gastrointestinal mucosa, potential teratogenicity, and drug resistance mechanisms [66].

Second-line agents, including PTM and AmB, are still being used as an option for treating infected patients; some of the most serious adverse effects caused by AmB are nephrotoxicity and myocarditis [28]. Moreover, liposomal AmB has been approved by the FDA in some countries as a better tolerated alternative when compared to conventional amphotericin B deoxycholate; however, due to its high cost, it has been limited as an option [29]. In addition, PTM has been used via systemic intravenous administration, its approval for use in certain types of leishmaniasis varies between countries, as the FDA-approved indications do not include leishmaniasis [6]; nevertheless, it shows insufficient efficacy, and its safety profile may include serious side effects such as renal toxicity and pancreatitis [25,29]. On the other hand, PMM is an antibiotic typically used to treat bacterial infections and has been used in topical formulations to treat selected cases of leishmaniasis. Topical and parenteral formulations have been approved in India as alternative treatments, but despite their low cost and absence of serious toxicity issues, some mechanisms related with drug resistance have been reported [25].

Among the strategies used to combat treatment failure in patients with leishmaniasis, combination therapies involving the use of different drugs in the same patient have been indicated; for instance, MLT has been used in combination with PMM and AmB as treatment. In India, a combination of AmB and MLT was used, resulting in a more cost-effective treatment [66]. However, a cross-resistance between these two drugs has been described by evaluating MLT-resistant mutants generated in vitro, and alterations in the lipid composition of the plasma membrane were found to also be associated with AmB resistance [67]. Combination therapies have also been used as a short-term treatment; for instance, combining MLT and PMM can result in a safe and highly effective alternative, suggesting that MLT could delay the expected development of resistance to PMM [68,69]. Although many strategies have been developed to provide access to treatment through public health programs in underdeveloped countries with high rates of the disease, there are other limitations, such as the high toxicity of certain drugs and the rapid development of drug-resistant parasites, which can lead to treatment failure. However, the molecular mechanisms of drug resistance are not well understood and require further investigation.

## 3. Genomic Changes and Drug Resistance

*Leishmania* has an atypical genome that displays an extreme plasticity, variation in chromosomal dosage between species, a lack of introns and the presence of a modified base [70,71,72]. The extreme plasticity is achieved by exploiting genome instability through gene dosage changes [70]. The genome is distributed over 34–36 chromosomes depending on the species. The different number of chromosomes between species seems to be associated with chromosome fusion as has been described in *L. braziliensis* and *L. mexicana* [71]. Furthermore, despite being a eukaryotic genome, it does not have introns and also transcribes polycistronically, like bacteria, although the polycistronic genes are not necessarily functionally related [73]. A modified thymidine (β-D-glucopyranosyloxymethyluracil) has also been found to be present in its genome, especially in the sub-telomeric regions and is known as the J base [74]. *Leishmania’s* genome has high plasticity, allowing it to develop genomic modifications as a survival mechanism in response to stressful conditions. These alterations can modify the expression levels of certain genes and play an important role in the parasite´s resistance to drugs commonly used to treat the disease. Some of those genomic alterations that affect or modulate gene expression levels are aneuploidies, genetic amplifications, and gene deletions [75,76,77,78]. These changes usually regulate the expression of drug targets, drug transporters, or enzymes associated with drug inactivation, but other modifications exist that are associated with specific changes in the sequence of a gene that modify or change the structure and/or function of proteins, as generally occurs with some single nucleotide polymorphisms (SNPs) [27,79]. In this review, the genomic changes associated with the most-used treatments for leishmaniasis will be discussed.

Polyploidy has been described for several chromosomes in several *Leishmania* species that are resistant to drugs [77,78,80,81,82], with a genomic amplification being one of the most common alterations leading to the change in the chromosomal number [80,83]. The most common genes that have been found to be involved in antimony resistance through changes in their ploidy are MRPA, APX, and G6PDH (Table 2) [80,83,84,85]. Changes associated with aneuploidies have been reported in PMM-resistant strains [81]. Gene deletion and mutations, especially deletions of nucleotides, also have been reported; these mutations may be alone or accompanied by gene deletions or gene amplifications, as is the case with the membrane transporter associated with antimony uptake, which is the AQP1 whose action has been associated with both antimonials and pentamidine [83]. Other mutations associated with antimonial-resistant species correspond to changes in the multi-drug resistance 1 (MDR1) protein [86]. On the other hand, in AmB-resistant parasites, an 8 Kb deletion in the gene that codes for 24-sterol methyltransferase (SMT) or mutations in sterol C5-desaturase (SC5D) or sterol 14-demethylase (CYP51) have also been reported [87,88]. Miltefosine (MLT) mutations in the MLT transporter gene have been described that have also been associated with cross-resistance to AmB; likewise, the deletion of the MLT-sensitive locus has been reported in four species of *Leishmania* [67,89,90]. In a study selecting the resistance to PMM in *Leishmania donovani* amastigotes, a total of 11 short nucleotide variations and alterations in copy numbers for 39 genes were associated with the resistance to this drug, several of which were involved in transcription, translation, and protein turnover [91]. The summary of the best studied genomic alterations associated with drug resistance is presented in Table 2.

Extrachromosomal DNA, which can be linear or circular, has also been detected in drug-resistant *Leishmania*. These episomal fragments have been found to be associated with resistance to antimony [76] and AmB [95]. An evaluation of *L. tarentolae* strains found that the more episomal copies the parasite had, the greater the resistance to AmB it exhibited [95]. Lately, the possibility of extracellular vesicles transferring from resistant to sensitive strains has been described, and the characterization of the vesicles´ content has demonstrated the presence of DNA containing genes associated with drug resistance [96].

## 4. Changes in Transcriptomes Associated with Drug Resistance

*Leishmania’s* dynamic genome plays a vital role in the development of drug resistance; however, the role of transcriptional control is limited due to its unique mechanisms. Contrary to most other eukaryotes, *Leishmania* parasites lack introns and employ unidirectional polycistronic transcription units (PTUs). From PTUs, pre-mRNA encoding potential polypeptides are produced, typically via RNA polymerase II (RNAP II). The transcription of the PTU by RNAP II is terminated upon the detection of the unique kinetoplast base J (β-D-glucosyl-hydroxymethyl uracil), first identified in *T. brucei,* located in the sub-telomeric regions, and later shown to prevent transcriptional readthrough in *Leishmania* [74,97,98,99]. Resulting polycistronic pre-mRNA are then processed into mature mRNA via two mechanisms: (1) trans-splicing with the addition of a 39–41 nucleotide spliced leader RNA to the 5′-terminus and (2) 3′-end cleavage/polyadenylation [100].

*Leishmania* parasites lack the canonical transcription factors, promoters, and individually regulated genes found in higher eukaryotes. This apparent lack of transcriptional regulation; however, seems to indicate that modulation occurs via a few different mechanisms: gene dosage variation and post-transcriptional regulation. Mechanisms of gene dosage modulation include the generation of episomal amplicons and mosaic aneuploidy, including regional and chromosomal copy number variation (CNV) [79]. Translational efficiency and mRNA stability are crucial for the modulation of effective transcript quantity. These mechanisms are not fully understood in the context of therapeutic resistance, but they nonetheless offer insight into the complex and interconnected methods by which *Leishmania* spp. are able to rapidly develop resistance [101].

Among the most studied antileishmanial therapeutic options, Sb^V^ have been shown to elicit profound modulatory effects at the mRNA level, likely due to the existence of multiple cellular targets [102]. Among these modulations, some of the most important differentially expressed genes (DEGs) in resistant *L. infantum* lines were found to play a role in protein phosphorylation, microtubule-based movement, protein ubiquitination, stress response (e.g., HSP-100 and DNAJ), the regulation of membrane lipid distribution (e.g., ABC transporters), RNA metabolism proteins (e.g., RNA-binding proteins (RBPs)), translation, and ribosome biogenesis [102]. In the same study, other notable over-expressions in the transcriptome included GSH1 (encoding γ-GCS, a key enzyme in the glutathione pathway), RBPs (particularly RNA recognition motifs responsible for transcriptional control), ribosomal proteins, ABC transporters (e.g., MRPA), and HSP-100 (a key component of the stress response). Other studies have shown similar expression patterns regarding these transcripts [87,103]. Notable in these studies was also a lack of differential expression for AQP1, a key drug-entry point which has previously been seen to be down-regulated in Sb^III^-resistant strains [23].

Another important consideration includes expression across varying life cycle stages and species. In a recent study, the transcriptome of *L. infantum* amastigotes resistant to antimonials displayed significant differential expression which contrasted with profiles found in the promastigotes of other studies and likely contributed to their survival within the harsh phagolysosome environment [104]. Another study found differential responses to Sb^III^ in a comparative analysis across five *Leishmania* species of medical importance, but found no common DEGs across these five species—although RBPs, nicotinamide adenine dinucleotide phosphate (NADPH), and the cytochrome-B5-oxidoreductase complex were found among four of the five [105].

Regarding MLT, resistance mechanisms at the mRNA level have been correlated with alterations in drug transport, modulating the MT/ROS3 transporter complex [106,107,108,109]. Likewise, it has been demonstrated that differential gene expression of the miltefosine transporter complex is involved in the parasite’s susceptibility to MLT [110]. Another study, however, found only a slight up-regulation of LMT/LROS3 (an accessory protein) in resistant *L. donovoni* parasites [87]. In this study, a heterozygous SNP in the *Leishmania* miltefosine transporter (LMT) gene, leading to the appearance of a stop codon potentially associated with decreased miltefosine transporter expression, was found, along with the down-regulation of the ribosomal protein L17, amastin-like surface proteins, and superoxide dismutase, as well as the up-regulation of histone H1. Additionally, comparative analyses of MLT-resistant and MLT-sensitive *L. donovani* transcriptomes have shown significant modulation of the following mechanisms: (i) DNA replication and repair machinery, (ii) protein translation, (iii) energy generation mechanisms, (iv) transporters (e.g., ABC1, ABCA7, and AQP1), and (v) antioxidant defense mechanisms [111]. Similar findings across these studies may suggest that MLT resistance in *Leishmania* spp. is more profoundly influenced by regulation elsewhere along the expression pathway.

Many of the other antileishmanial agents have also been shown to exert a modulatory effect on *Leishmania* spp. transcriptomes but are less studied than Sb^V^ or MLT. In regards to AmB, a study of four resistant *L. mexicana* lines demonstrated reduced expression of two genes coding for sterol C24-methyltransferase (SMT) influenced by chromosomal ploidy, which is associated with the resistance to this drug in multiple strains [94]. These observations were mirrored in another study of resistant *L. donovani*, which additionally showed the up-regulation of amastins and tryparedoxin 1 transcripts (an antioxidant molecule which has been previously linked to AmB resistance) [87,112]. Transcript levels of Sir2 and the related PARP1 (apoptotic regulators) have also been associated with AmB resistance in *L. donovani*, demonstrating effects on drug efflux, the ABC transporter (MDR1) mRNA levels, and ROS concentrations [113]. As for PMM, transcriptome profiling of PMM-resistant *L. donovani* suggests adaptations including: (i) the down-regulation of the aerobic metabolism, (ii) the up-regulation of glycolysis and glycosomal succinate fermentation, (iii) decreased DNA synthesis (via the down-regulation of DNA polymerase θ) and increased DNA repair, (iv) decreased protein synthesis and degradation (the down-regulation of tryptophanyl-t-RNA synthetase, ribosomal proteins, metallopeptidases, and carboxypeptidases), and (v) increased PMM efflux by means of the increased expression of ABC transporters [114]. Another study, also using PMM-resistant *L. donovani*, found DEGs including two very strongly up-regulated transcripts encoding D-lactate dehydrogenase-like protein (D-LDH) and the aminotransferase of branched-chain amino acids (BCAT) [87]. This study also suggested that the overexpression of these proteins may allow for alternative energy production in the form of additional ATPs to compensate for a mitochondrial dysfunction, as well as the up-regulation of proteins including ABC10, ribosomal protein L38, and amastins.

## 5. Translational Control as a Major Driver of Drug Resistance

*Leishmania* spp. eludes transcriptional control due to the nature of its polycistronic transcription and the absence of other elements important for the regulation of RNA levels at pre-translational stages, such as RNA interference (RNAi) machinery [100]. In contrast, other eukaryotes have monocistronic transcription, meaning that there is only one gene per mRNA and introns are removed from pre-mRNA within the same transcript. Furthermore, promoters, enhancers, and other regulatory elements interact with the translation machinery to control transcription initiation, referring transcriptional and post-transcriptional control the dominant mode of gene regulation [115,116]. This behavioral pattern of *Leishmania* spp. is also exhibited in other trypanosomatids, such as *T. brucei* and *T. cruzi*, since their genetic regulation seems to primarily be led by post-transcriptional mechanisms such as mRNA stability, translational control, and RNA-binding proteins (RBPs) [100,101].

This absence of transcriptional regulation suggests that parasites require more specialized translational machinery as a compensatory mechanism to maintain mRNA stability and regulate gene expression [117]. Translational control mechanisms, such as the altered initiation, elongation, or termination of mRNA translation, can directly impact the dynamic abundance of proteins crucial to the parasite response to varied stimuli [115]. However, the role of translational control in drug resistance has been poorly investigated. It has been shown previously that calcium-dependent protein kinase 1 (CDPK1) acts as a modulator of translation efficiency for selective transcripts, and mutations in this protein contribute to paramomycin and antimony resistance [118]. This kinase can alter the translational efficiency of mRNAs encoding for drug efflux pumps and enzymes involved in drug metabolism, thereby enhancing the parasite’s ability to survive in the presence of therapeutic agents and hinting at the importance of translational control in drug resistance in *Leishmania* spp. [118,119].

**Figure 3:**
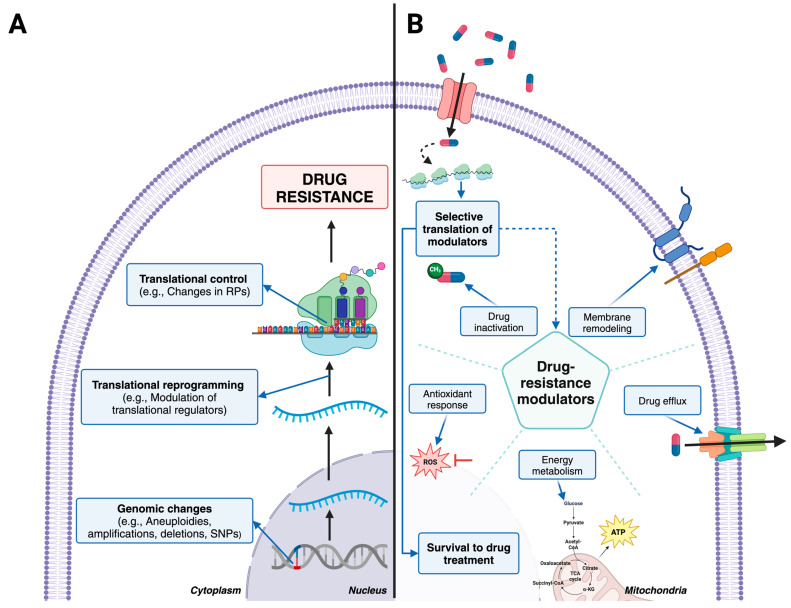
Molecular mechanisms of drug resistance in *Leishmania. (***A**) Mechanisms employed by *Leishmania* to develop drug resistance involve changes at the genomic and translational levels. Resistant parasites possess a remodeled translational profile that is distinct from profile of sensitive parasites even in the absence of the antimony drug, which serves as a pre-emptive adaptation to drug challenge. (**B**) This depicts the response of drug-resistant parasites to drug challenge. Once the resistant parasite recognizes the presence of the drug, it activates the selective translation of mRNAs encoding drug-resistance modulators. These modulators are involved in various response mechanisms, such as surface protein remodeling, drug efflux, the optimization of the energy metabolism, antioxidant response, and drug inactivation. Together, these mechanisms act quickly and in a coordinated manner to combat treatment.

In order to investigate the role of translational control in drug resistance, our group examined translatomes of sensitive and resistant *Leishmania tropica* parasites. Resistant parasites were generated using parental sensitive parasites through stepwise antimony selection [28]. We have shown that the development of antimony resistance involves a dramatic reprogramming of mRNA translation in *Leishmania* parasites (Figure 3). Translatomes of resistant parasites were drastically different from those of sensitive parasites, even in the absence of the antimony drug, and included 2431 differentially translated transcripts (DTTs). The transcriptome analysis demonstrated that the vast majority of changes in resistant parasites are observed in the translatome rather than the transcriptome, supporting the important role of translational control in drug resistance. In contrast, resistant parasites that are grown while exposed to antimony induced changes in the translation of a specific population of only 189 transcripts. Dramatic changes in the translatome observed in the absence of the drug challenge support that complex pre-emptive adaptations are needed to compensate for the loss of the biological fitness of parasites exposed to the drug essential for immediate survival to the drug challenge. This reprogramming includes the translation of mRNAs involved in trypanothione metabolism (a defense mechanism against oxidative stress), proteins related to drug efflux, and the remodeling of the cell membrane. Likewise, an enrichment of genes involved in the energy metabolism and the expression of putative translational regulators is also observed. Thus, the coordinated reprogramming of translation leads to diverse pre-emptive adaptations that combat drug effects and involve enhanced antioxidant response, energy metabolism, and cell surface, lipidome, and metabolome remodeling [28,120,121]. Our recent findings in the same drug-resistant *Leishmania tropica* parasites uncovered dramatic lipidome and metabolome remodeling, even in the absence of the antimony drug [120,121]. Our data support that those changes could be essential pre-emptive adaptations needed to counteract the drug upon exposure. We hypothesize that during the stepwise development of drug resistance, parasites exhibit changes in the translatome directed by translational regulators. The translational regulators themselves undergo changes occurring at both genomic and translational levels to support the complex reprogramming of translation. The association between drug-resistant phenotypes and reprogrammed mRNA translation has been previously observed in refractory cancer cells [122]. Translational reprogramming is commonly recognized as a source of adaptive plasticity that allows cancer cells to become resistant to new therapies and our data support that this is the case in *Leishmania* parasites.

## 6. Changes in Metabolomes and Lipidomes Associated with Drug Resistance

Drug-resistant parasites are known to exhibit profound changes in both the lipidome and metabolome to support drug resistance [120,121]. Metabolomic approaches have unraveled many of the mechanisms of action of drugs used to combat *Leishmania* infections; additionally, they have demonstrated how parasites can respond to these drugs and the strategies they use to acquire resistance [123,124,125,126,127,128]. This resistance involves alterations and the differential expression of metabolites involved in lipid, energy, or amino acid metabolism. Nonetheless, the metabolic mechanisms used by resistant parasites are still poorly understood, with lipid remodeling and changes in lipid metabolism being the most characterized so far.

At the lipidome level, *Leishmania* parasites have exhibited changes in the composition of phospholipids, fatty acids, sphingolipids, and glycerolipids in drug-resistant strains. For instance, fatty acids, the most prominent type of fatty acyls and the building blocks for several lipids [129], have long and highly unsaturated alkyl chains in different membrane components like phospholipids, glycerolipids, and glycolipids in drug-resistant parasites [29]. These changes have been reported in *Leishmania* strains resistant to MLT [130], AmB [131], and Sb^V^ [80,120,132], and it has been suggested that these alterations can decrease the ordered state of the membranes and modulate their fluidity, thereby improving the parasitic response to drug treatment and the resistance to oxidative stress [29,130,131,133]. On the other hand, phosphatidylcholines (PCs) and phosphatidylethanolamines (PEs), two of the most abundant phospholipids (PLs) in the cell membranes of *Leishmania*, have exhibited alterations in abundance and composition in resistant parasites [51,134,135]. PCs with high fatty acyl unsaturation are more abundant in Sb and MLT-resistant strains, usually accompanied by changes in the quantity of PEs and other phospholipids [29,120,123]. Sterols are another group of altered lipids in resistant strains of *Leishmania*. Ergosterol and ergosterol-like lipids are the main sterols in the cell membranes of trypanosomatids, while cholesterol (despite not being synthesized by *Leishmania*) is incorporated from the environment [136,137]. Some studies have shown differences in the sterol composition of resistant parasite membranes. For instance, increased levels of ergosterol have been observed in Sb^III^-resistant parasites [138] and cholesta-5,7,24-trien-3β-ol (instead of ergosterol) is increased in AmB-resistant strains [131], but reduced sterol biosynthesis is observed in atovaquone and MLT-resistant strains [130,139]. On the other hand, cholesterol is increased in MLT-resistant parasites [139], while both cholesterol and ergosterol are reduced in sitamaquine-resistant *Leishmania* [140]. Other altered lipids found in drug-resistant strains of *Leishmania* are sphingolipids, being generally reduced in Sb-resistant strains [29,120,123,127].

Regarding the amino acid metabolism, some studies have shown differences in the abundance of metabolites relevant for cell survival during environmental stress in drug-resistant parasites. One such metabolite is proline, an essential amino acid mainly used as a carbon source for recovery during the osmotic stress response and as a protective agent during oxidative stress [141]. Proline is highly abundant in Sb-resistant parasites [80,121,123,127,142], but is not involved in PMM resistance [81]. Similar to proline, alanine is an amino acid contributing to osmotic balance in *Leishmania* spp. which is increased in Sb-resistant parasites [121,127,142]. Arginine is another important amino acid in resistant strains, involved in the polyamine metabolism and activating trypanothione downstream [143]. Arginine has been found to be elevated in Sb-resistant parasites [80,121,142]. The increase in these amino acids, accompanied by the decrease in others like betaine, helps resistant parasites respond to oxidative and osmotic stress while potentially even inducing drug inactivation by thiol metabolism [121].

Energy metabolism is also important in resistant parasites since it contributes to energy production by fueling the NADPH necessary for drug response pathways like thiol metabolism activation [121]. Some metabolites involved in the energy metabolism of drug-resistant parasites are acetate, valine, threonine, and lactate [80,81,121,127,142].

All of these that are coordinated in metabolomes and lipidomes are essential for the development of drug resistance and *Leishmania* spp. survival. Drug resistance is a multifactorial chain of events that is still not fully understood in *Leishmania* spp. However, the study of resistant strains through different molecular approaches may help to clarify the unique mechanisms used by these parasites to develop resistance and illuminate the path to finding new drug targets for combating leishmaniasis.

## 7. Conclusions and Perspectives

In this review, we have summarized known molecular mechanisms by which *Leishmania* spp. may develop drug resistance and evaluated the main mechanisms in protozoa parasites and other eukaryotes during the gene regulation associated with drug resistance. Genomic alterations, such as aneuploidies, gene deletions, and SNPs, could modify gene expression and protein function, particularly in response to commonly used treatments like Sb, AmB, MLT, and PMM. Transcriptomic control, while limited, further reveals the parasite’s ability to adapt to drug pressures through the differential expression of genes involved in several cellular processes, including stress responses, protein ubiquitination, and RNA metabolism. Otherwise, due to the lack of transcriptional regulation, the importance of translational control mechanisms in *Leishmania* spp. highlights the role of mRNA stability and specialized translational machinery in drug resistance. Some key proteins, such as CDPK1 and ABC transporters, are crucial to the modulation of a parasite’s response to drugs, and metabolic changes coordinated during translational reprograming also support resistance by enhancing the parasite’s ability to face oxidative stress and maintain membrane integrity.

Despite the extensive research that has already been conducted, significant gaps exist in understanding the full picture of drug resistance in *Leishmania* parasites. The interaction between genomic changes and translational control as well as how both processes influence resistance mechanisms are still unclear. The variability in the regulation of resistance mechanisms between different *Leishmania* species, different life cycle stages, and throughout the infection processes also require more exploration. Furthermore, the precise mechanisms of translational control and their contributions to resistance, like the role of translational reprogramming in pre-emptive adaptations, require further investigation through the validation of genes associated with resistance using CRISPR-Cas9 functional gene knock-out analysis. It remains unknown if similar translational reprogramming mechanisms exist in parasites that are resistant to different drugs and what differences parasites exhibit in response to different drugs at a translational level. It is important to carry out more work on naturally occurring resistant strains obtained from patients who did not respond to treatment. With so much still being unknown, there is significant motivation to continue the study of drug resistance in *Leishmania* spp. using integrative advanced sequencing technologies, polysome profiling, proteomics, and other novel techniques [28,120,144]. Investigating translational modulation could identify new gene targets and regulatory elements involved in the drug resistance pathways. Exploring the role of different proteins in specific translational control pathways or the stress response could lead to promising strategies to combat drug resistance in *Leishmania* spp. Lastly, investigating the role of non-coding RNAs during the regulation of gene expression and translation could provide new pharmacological targets for the development of novel therapeutics.

## Figures and Tables

**Table 1:** Modes of action of current antileishmanial drugs.

Current Drugs for Leishmaniasis Treatment	Mode of Action and Parasite Targeting	References
**Pentavalent** **Antimony** **(Sb^V^)**	Inhibits the mitochondrial enzyme trypanothione reductase, increasing the parasite’s susceptibility to oxidative stress generated by the macrophage during infection. It can obstruct major energy-driven pathways such as fatty acid oxidation and glycolysis.	[35,48,49]
**Miltefosine** **(MLT)**	Inhibits the enzyme cytochrome c oxidase located in the mitochondria, directly affecting energy production in the parasite. Also inhibits phosphatidylcholine synthesis, which affects lipid metabolism through the CDP-choline pathway by acting on CTP-phosphocholine cytidylyltransferase activity.	[50,51]
**Liposomal** **amphotericin B** **(AmB)**	Forms transmembrane channels through the cell wall and is known to have a high affinity for ergosterol, causing micropores in the membrane, increasing permeability and ion loss, and resulting in cell death.	[52,53]
**Paromomycin** **(PMM)**	Inhibits the cytosolic ribosome, affecting protein synthesis through binding to the 16S ribosomal unit and creating an alteration in its structure.	[54,55,56]
**Pentamidine** **(PTM)**	Inhibits DNA and protein synthesis and causes cell-cycle arrest in the G2/M phase. Inhibits RNA polymerase, leading to apoptosis. Inhibits arginine transport.	[57,58]

**Table 2:** Genomic alterations associated with drug resistance.

Drug	Gene Name	Genomic Changes	Effect Associated with Drug Resistance	Reference
**Antimony**	MRPA	Amplification	Increases drug efflux	[80,92]
	APX	Amplification	Protection from ROS accumulation	[84]
	G6PDH	Amplification	Protection from ROS accumulation	[84,93]
	AQP1	Amplification, Deletion	Reduces drug uptake	[83]
	MDR1	Point mutation	Increases drug efflux	[86]
**AmB**	SMT	Deletion	Reduces drug uptake	[87]
	SC5D	Point mutation	Alters sterol biosynthesis	[94]
	CYP51	Point mutation	Alters sterol biosynthesis	[88]
	LMT	Deletion, Point mutation	Alters sterol biosynthesis	[67]
**Miltefosine**	LMT	Deletion	Reduces drug uptake	[89]
**Paromomycin**	Gene 18S RNA	Point mutation	Decreases binding of PMM	[81]

## Data Availability

Not applicable.

## Abbreviations

aa-tRNA	Aminoacyl transfer ribonucleic acid
ABC	ATP binding cassette
AmB	Amphotericin B
APX	Ascorbate Peroxidase
AQP1	Aquaglyceroporin 1
ATP	Adenosine triphosphate
BCAT	Branched-chain amino acids
CDP	Cytidine-5’-diphosphate
CNV	Copy number variation
CTP	Cytidine-5’-triphosphate
CDPK1	Calcium dependent protein kinase 1
CL	Cutaneous Leishmaniasis
CRISPR-Cas9	Clustered Regularly Interspaced Short Palindromic Repeats-Protein 9
CYP51	Sterol 14-demethylase
D-LDH	D-lactate dehydrogenase-like protein
DNA	Deoxyribonucleic Acid
DEGs	Differentially expressed genes
FDA	Food and Drug administration
G6PDH	Glucose-6-Phosphate Dehydrogenase
HAPT1	High affinity pentamidine transporter
HIV	Human immunodeficiency virus
JAK-STAT	Janus kinase/signal transducers and activators of transcription
LMT	Miltefosine Transporter gene
MCL	Muco-Cutaneous Leishmaniasis
MDR1	Multi-drug resistance 1
MLT	Miltefosine
mRNA	Messenger ribonucleic acid
MRPA	Multidrug-resistance protein A
NADPH	Nicotinamide adenine dinucleotide phosphate
NTD	Neglected tropical disease
PC	Phosphatidylcholines
PE	Phosphatidylethanolamines
PL	Phospholipids
PMM	Paromomycin
PRP1	Pentamidine resistance protein 1
PTM	Pentamidine
PTUs	Polycistronic transcription units
PDR-1	Pectin degradation regulator-1
RBP	RNA Binding protein
RBPs	RNA-binding proteins
RNAP II	RNA polymerase II
RPP	Ribosomal protection protein
rRNA	Ribosomal ribonucleic acid
Sb^V^	Pentavalent antimony
SC5D	Sterol C5 -desaturase
SMT	24-sterol methyltransferase
SNPs	Single nucleotide polymorphisms
VL	Visceral Leishmaniasis
